# Supplementary material for: Molecular mechanism of plasmid-borne resistance to sulfonamide antibiotics
Source: Nat Commun. 2023 Jul 7;14:4031. doi: 10.1038/s41467-023-39778-7 (PMC10328974; doi:10.1038/s41467-023-39778-7)
Supplement: Supplementary file 1 — Supplementary Information [file 41467_2023_39778_MOESM1_ESM.pdf]

SUPPLEMENTARY FIGURES

Fig. S1. Chemical structures of sulfonamide compounds.

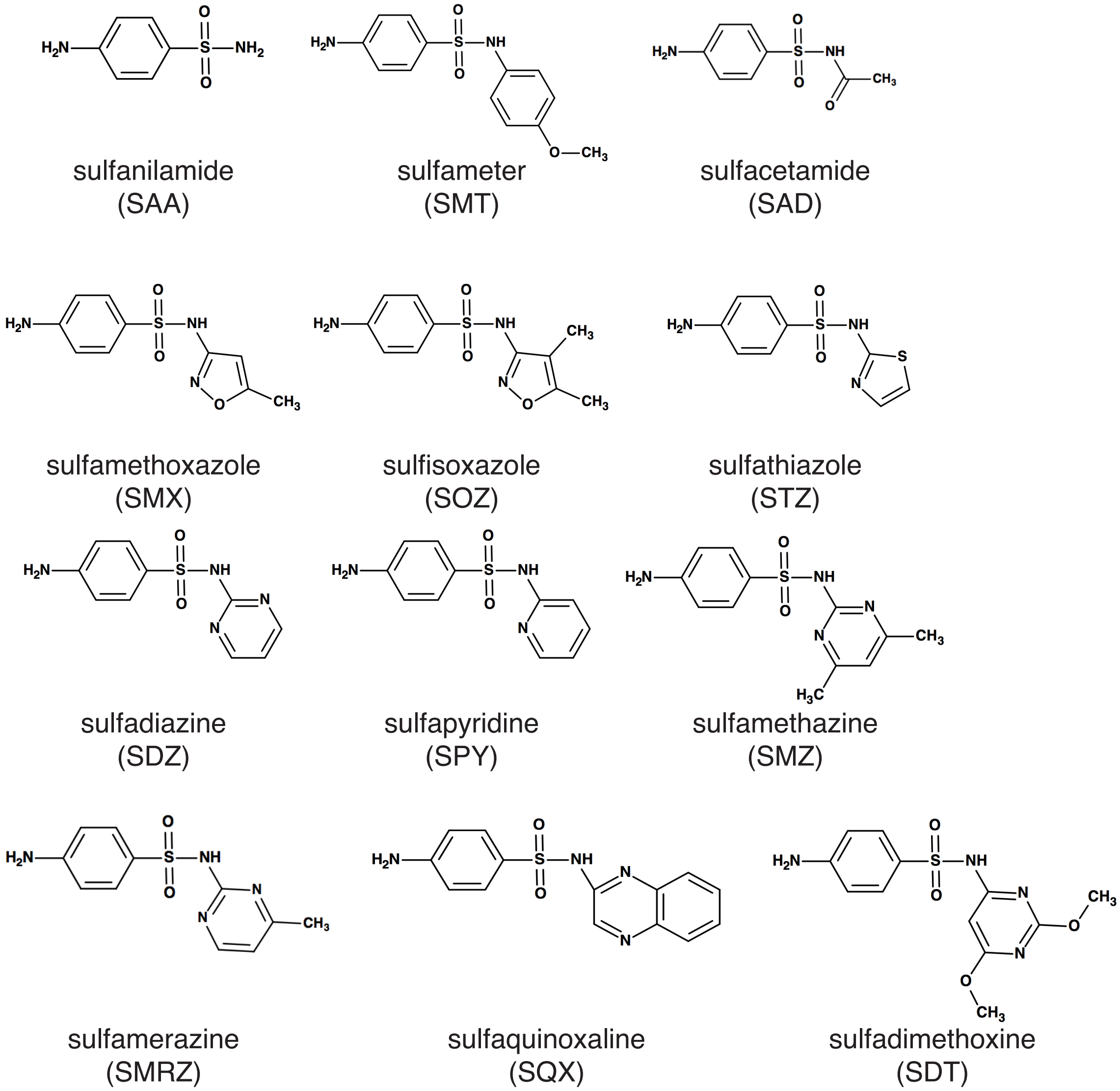

Fig. S2. Michaelis-Menten kinetics plots for *p*ABA for Sul and *Ec*DHPS enzymes.

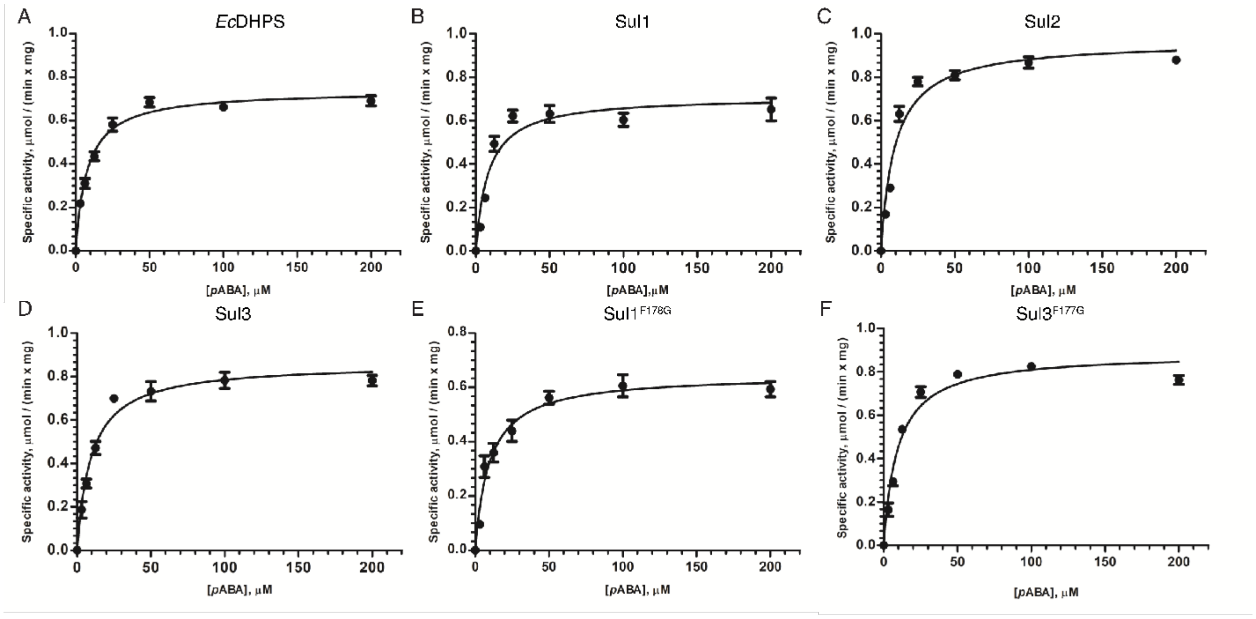

Shown are specific activities for (A) *Ec*DHPS, (B) Sul1, (C) Sul2, (D) Sul3, (E) $Sul1^{F178G}$, (F) $Sul3^{F177G}$ for the substrate *p*ABA in the presence of saturating excess of DHPP at 200 µM. The kinetic parameters K_M and V_{max} were estimated using the Michaelis-Menten equation by non-linear regression using GraphPad Prism v5.0. Each data point is the mean of three biological replicates for each substrate concentration. Each replicate is plotted as mean ± SD. Source data are provided in Source Data.zip.

Fig. S3. Michaelis-Menten kinetics and inhibition plots for SMX for Sul and *Ec*DHPS enzymes.

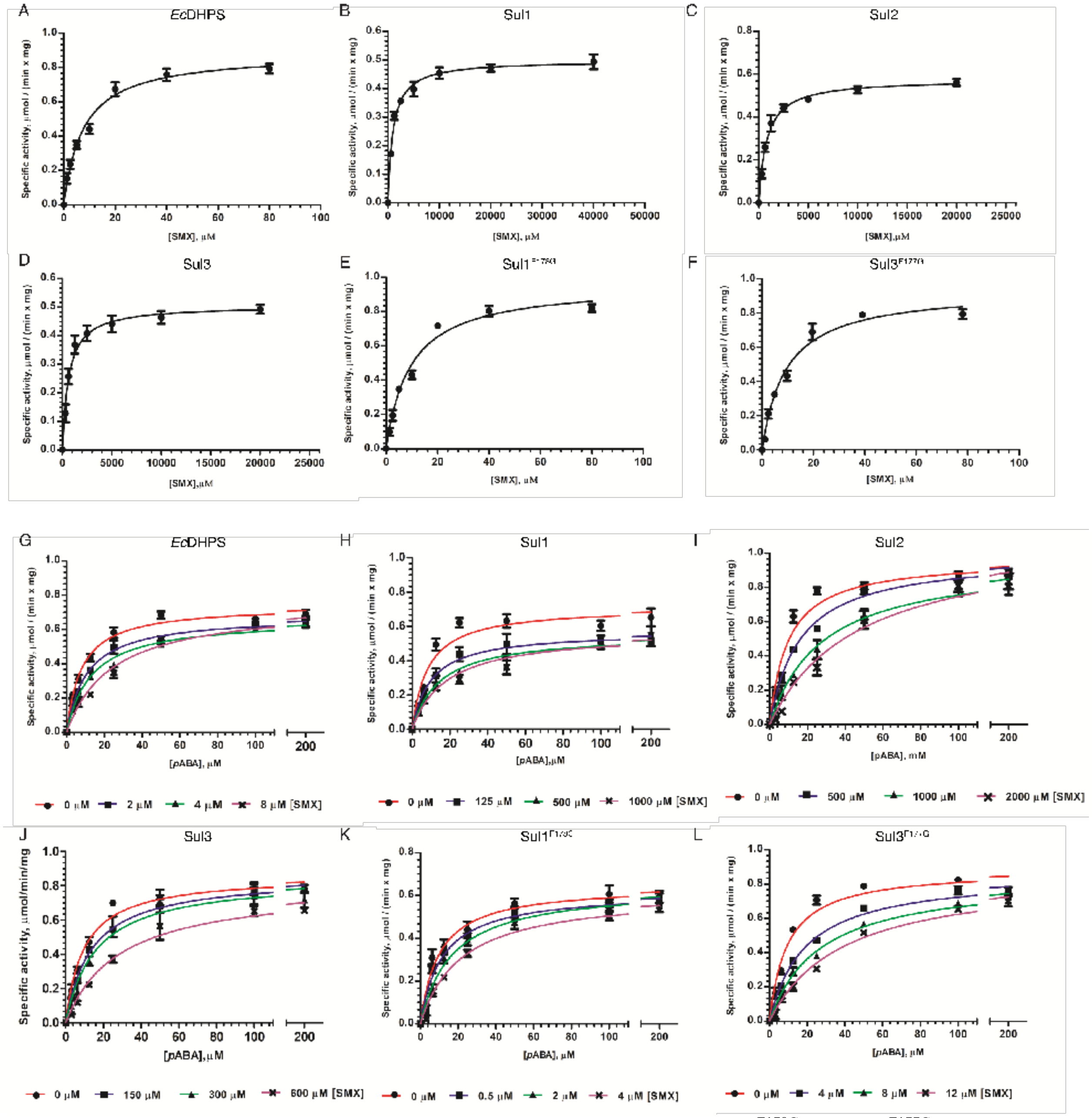

Michaelis Menten plots for (A) *Ec*DHPS, (B) Sul1, (C) Sul2, (D) Sul3, (E) $Sul1^{F178G}$, (F) $Sul3^{F177G}$ for the substrate SMX in the presence of saturating excess of DHPP at 200 µM. The kinetic parameters K_M and V_{max} were estimated using the Michaelis-Menten equation by non-linear regression using GraphPad Prism v5.0. For calculations of inhibition constant (K_i) for SMX for Sul enzymes: Michaelis Menten plots for (G) *Ec*DHPS, (H) Sul1, (I) Sul2, (J) Sul3, (K) $Sul1^{F178G}$, (L) $Sul3^{F177G}$ with varying SMX concentrations, in the presence of saturating excess of DHPP at 200 µM. Inhibition constant K_i for SMX was calculated using the formula as described in the *Experimental*. Each data point is the mean of three biological replicates for each substrate concentration. Each replicate is plotted as mean ± SD. Source data are provided in Source Data.zip.

Fig. S4. Mass spectrometry detection of formation of SMX-pterin adduct catalyzed by *Ec*DHPS and Sul1.

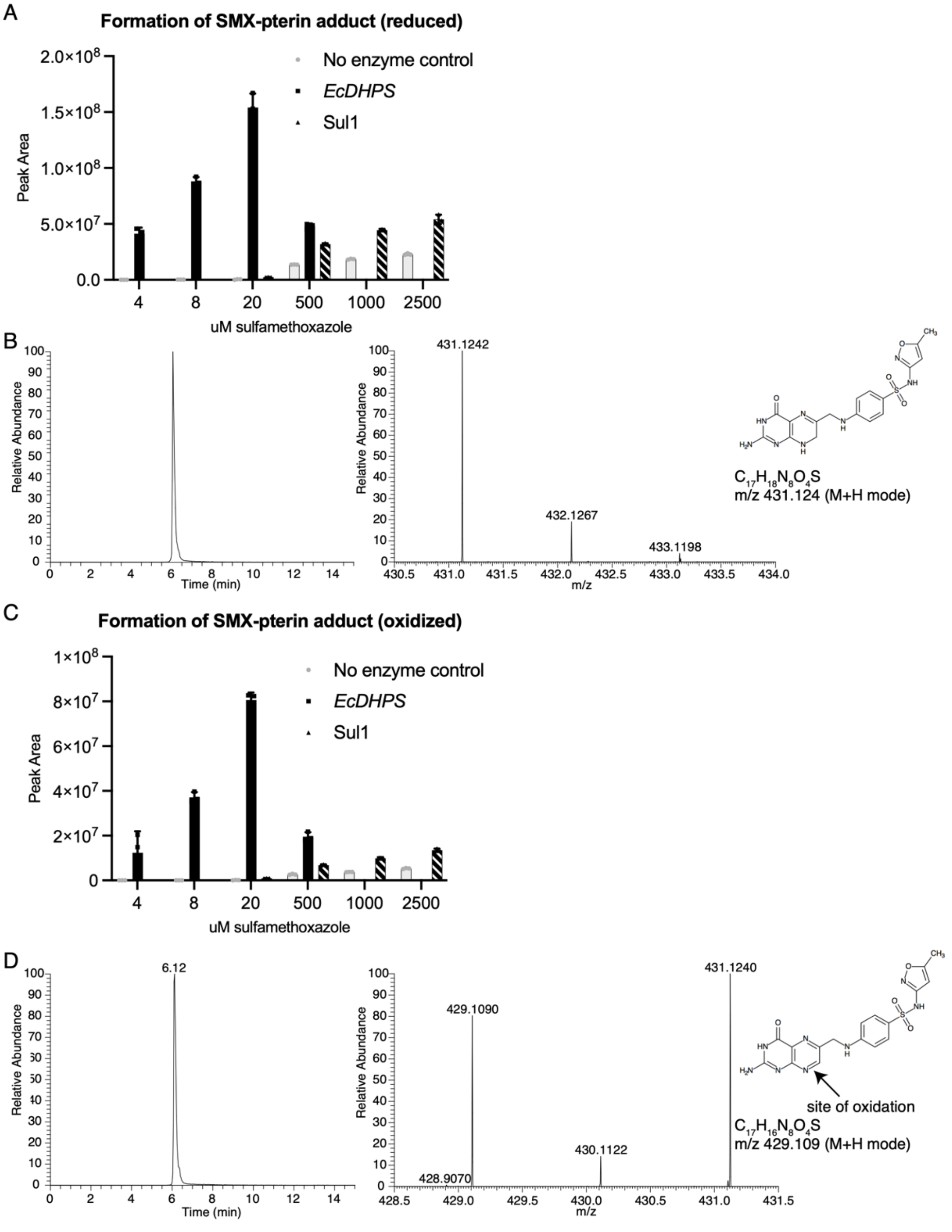

(A) Comparison of peak areas corresponding to reduced sulfamethoxazole (SMX)-pterin adduct species, indicated on the right of panel B, from mixtures of *Ec*DHPS (solid black bars), Sul1 (hatched bars) and no enzyme control (grey bars) plus excess DHPP, and the indicated concentrations of sulfamethoxazole. Biological triplicate data, mean and standard deviation shown.

(B) Extracted ion chromatogram (left) for the reduced SMX-pterin [M+H]+ ion (m/z 461.1242) with a 5 ppm error window, and representative mass spectrum with inlayed structure (right).

(C) Comparison of peak areas corresponding to oxidized sulfamethoxazole (SMX)-pterin adduct species, indicated on the right of panel B, from mixtures of EcDHPS (solid black bars), Sul1 (hatched bars) and no enzyme control. Biological triplicate data, mean and standard deviation shown.

(D) Extracted ion chromatogram (left) for the oxidized SMX-pterin [M+H]+ ion (m/z 429.1090) with a 5 ppm error window, and representative mass spectrum with inlayed structure (right). Source data are provided in Source Data.zip.

Fig. S5. Multiple sequence alignment of Sul and selected DHPS enzymes.

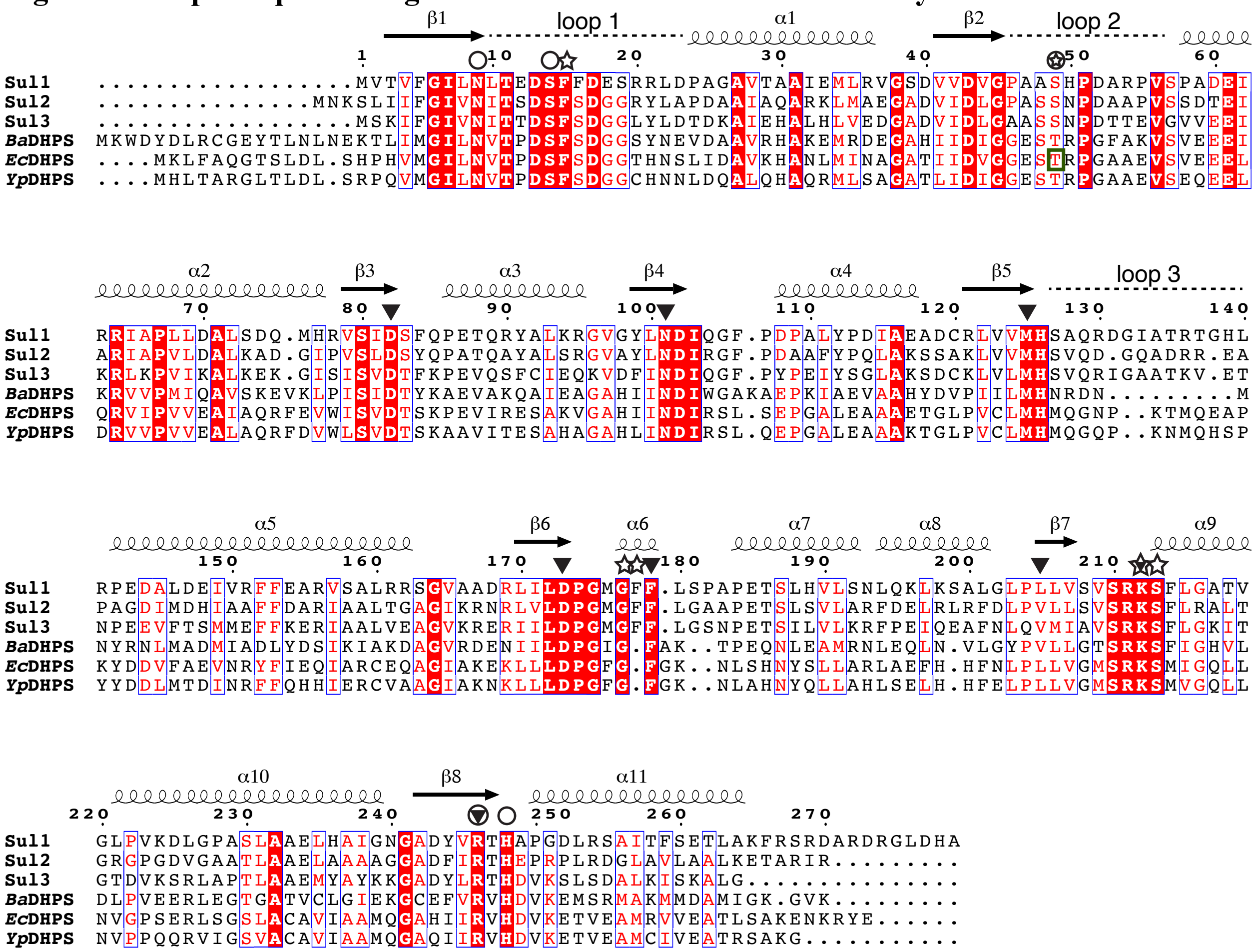

▼ interactions with pterin region

☆ interactions with *p*ABA

○ interactions with Mg^{2+} or PP_i

Secondary structure is indicated above the alignment. Positions interacting with ligands are labeled with triangle, star or circle as indicated in the legend. Shading is according to conservation, with white text/red background indicating complete sequence identity. Position 72 in *Ec*DHPS identified in adaptive laboratory evolution experiment is located in loop 2 and boxed in green. Source data are provided in Source Data.zip.

Fig. S6. Electron density maps of ligands bound to Sul and *Ec*DHPS crystal structures.

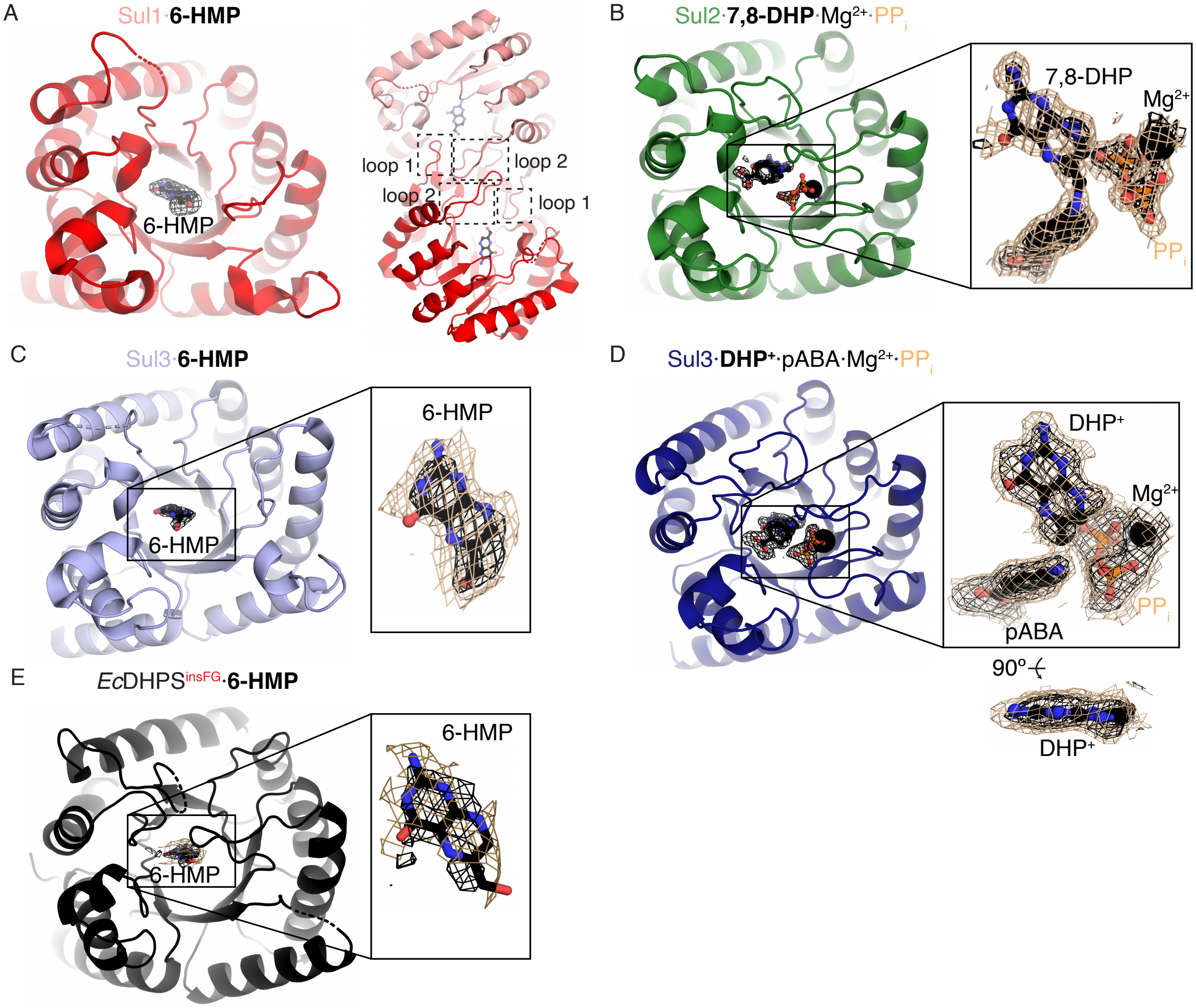

Electron density maps for ligands shown are simulated annealing omit maps (with ligand atoms and all atoms within 5 Å of the ligands deleted) contoured at 3.0σ (black) and 1.0σ (tan). (A) Sul1·6-HMP complex. Right = asymmetric unit showing interditigation of loops 1 and 2 into the other subunit's active sites. (B) Sul2·7,8-DHP·Mg^{2+}·PP_i complex. (C) Sul3·6-HMP complex. (D) Sul3·DHP^+·Mg^{2+}·PP_i complex, with 90º rotated view of DHP^+. E) *Ec*DHPS^{insFG}·6-HMP complex. Zoom-in show ligands in active sites.

Fig. S7. Comparison of pterin, magnesium and phosphate-interacting residues between Sul2, Sul3 and selected DHPS enzymes, and α6 Phe+loop 3 interactions.

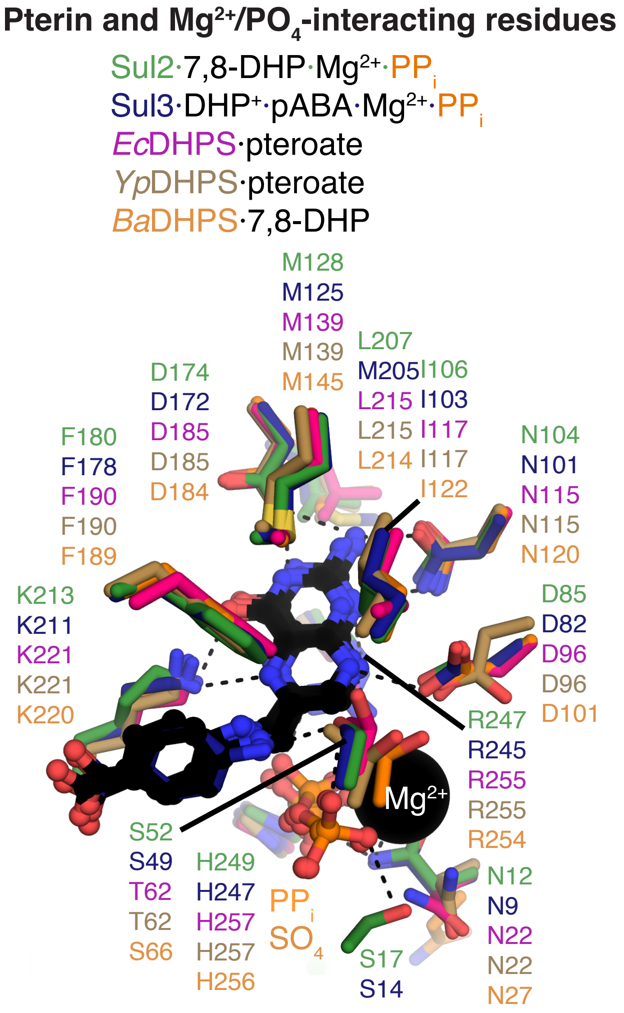

Shown are residues forming electrostatic, hydrogen-bonding or hydrophobic interactions with the pterin rings of bound ligands, magnesium ion, or phosphate/sulfate ions bound to the structures of Sul1, Sul3, *Ec*DHPS (PDB 5u10), *Yp*DHPS (PDB 3tyu) or *Ba*DHPS (PDB 3tya). All residues are identical except for Ser/Thr (i.e. Sul2 Ser52) which interacts with a phosphate, and a Leu/Met (i.e. Ser2 Leu207) which interacts with the pterin ring. Source data are provided in Source Data.zip.

Fig. S8. Modeling of sulfa drugs into the active site of Sul2.

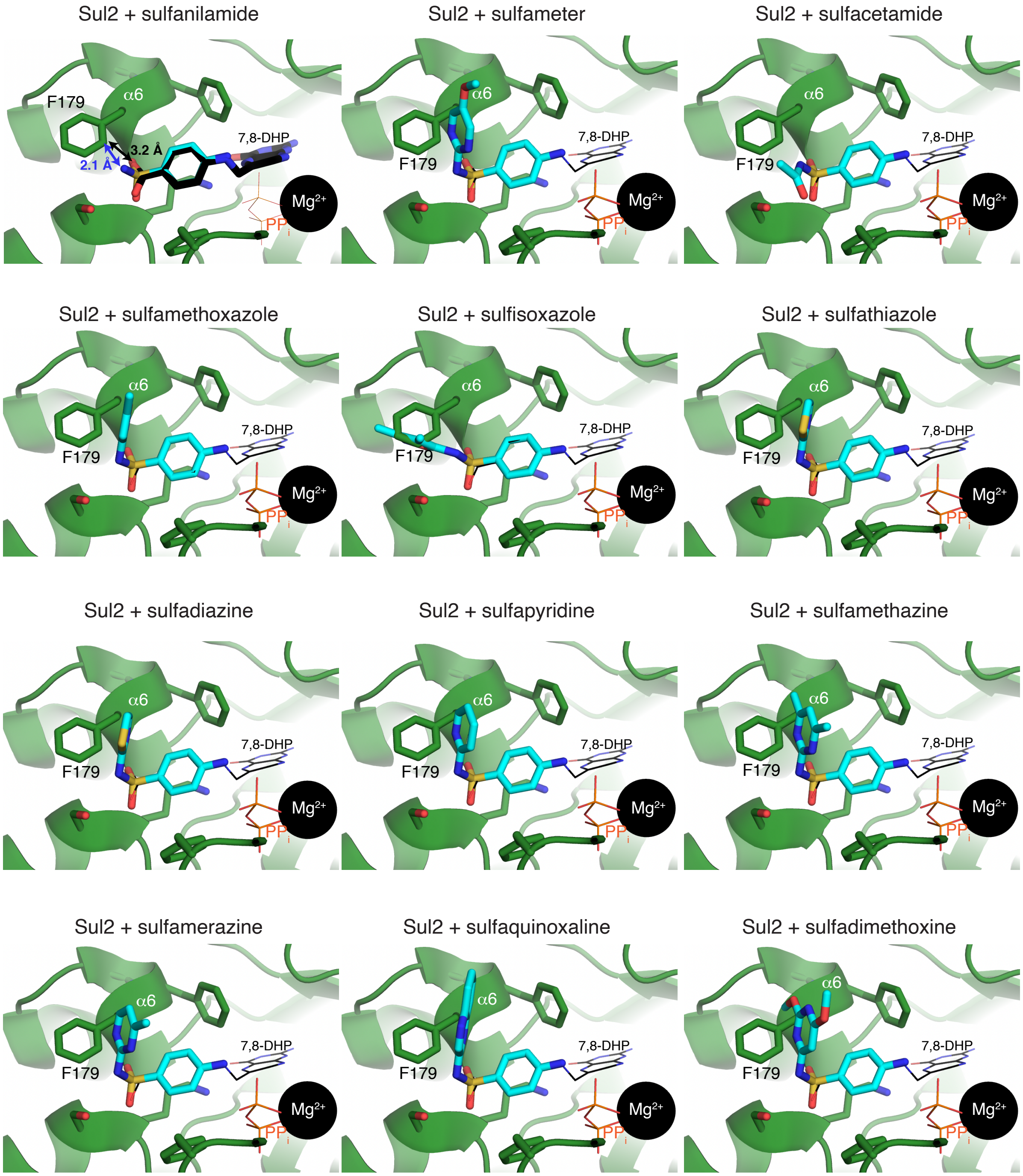

Models of the sulfa compounds (shown in cyan sticks) in the active site of Sul2. The 3D conformers (already energy minimized) of the 12 sulfonamide drugs were retrieved from Pubchem. Each of their 4-aminobenzene groups were superimposed onto the pABA region of 7,8-dihydropteroate from the Sul2·7,8-DHP·Mg^{2+}·PP_i complex structure. 7,8-DHP and PP_i from the Sul2·7,8-DHP·Mg^{2+}·PP_i complex are shown in thin lines, except for the sulfanilamide panel where 7,8-DHP is shown in thicker sticks. Mg^{2+} shown as a black sphere. F179 and α8 are labeled. The distance between the sulfonamide nitrogen (present in all sulfas) and the closest atom of F179 is indicated with a blue double-arrow and blue text (2.1 Å) on the sulfanilamide panel. The distance between the carboxylate oxygen of 7,8-DHP and the closest atom of F179 is indicated with a black double-arrow and black text (3.2 Å) on the sulfanilamide panel. Source data are provided in Source Data.zip.

Fig. S9. Intrinsic tryptophan fluorescence of Sul variants.

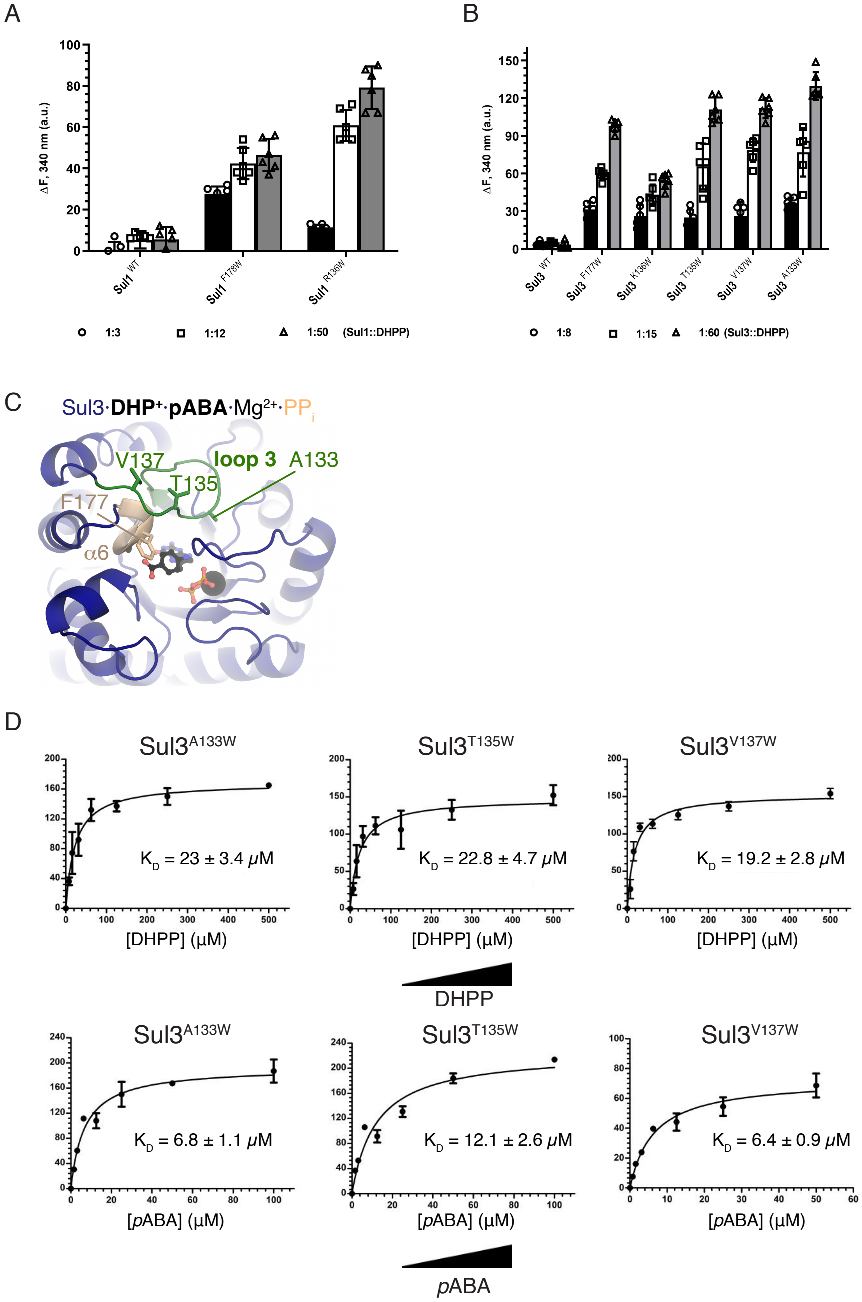

Maximum substrate-induced intrinsic tryptophan fluorescence (ITF) intensity changes monitored at 340 nm emission wavelength for (A) Sul3 and (B) Sul1 variants bound to substrate DHPP. The WT Sul1 and Sul3 enzymes do not have a Trp residue and therefore, show negligible baseline fluorescence intensity changes. Excitation wavelength was set as 295 nm (where the molar absorptivity of Trp is maximum while those of Phe and Tyr residues are low) and emission wavelength was monitored at 340 nm. Error bars represent ±SD. Bar graphs with overlay of corresponding data points are shown as scattered dot plots. The measure of each bar graph represents the mean value ±SD. The mean value represents data taken from two independent biological replicates performed in technical replicates. C) Location of Trp mutation sites in the structure of Sul3. D) Substrate binding affinities (K_D) of Sul3 Trp mutants for DHPP and *p*ABA. Substrate-induced fluorescence emission intensity change at 340 nm was plotted against substrate concentrations to derive the K_D by GraphPad Prism v5.0. Triplicate readings were average, and K_D reported as (±SD). Data from two biological replicates performed in technical replicates were used and K_D is reported as (±SD). Source data are provided in Source Data.zip.

Fig. S10. Intrinsic tryptophan fluorescence of *Ec*DHPS variants.

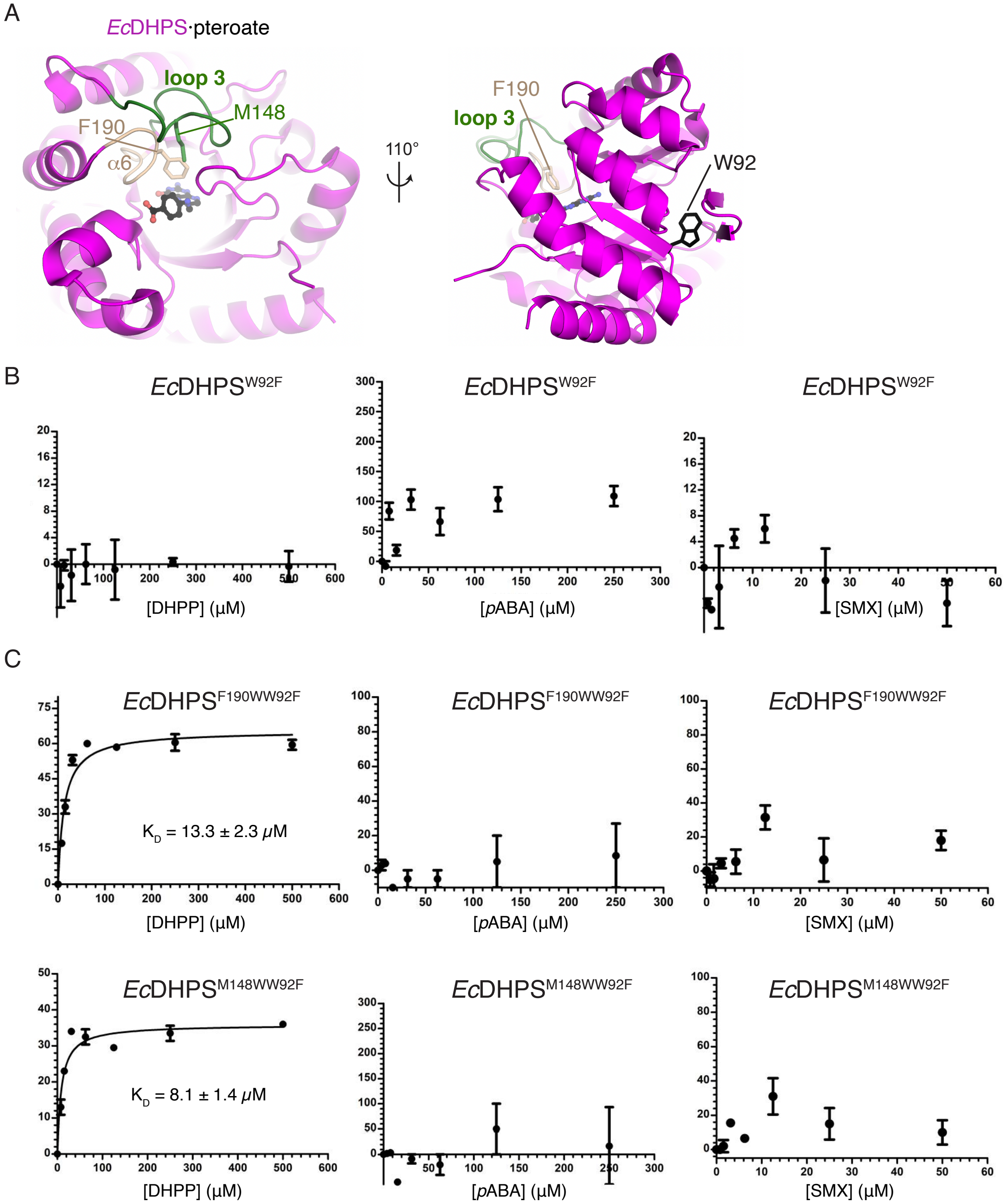

(A) Location of Trp mutation sites (F190, M148) and natural W92 residue in the structure of *Ec*DHPS. (B) Validation of no change in tryptophan fluorescence of *Ec*DHPS^{W92F} in response to DHPP, *p*ABA or SMX. Excitation wavelength was set as 295 nm (where the molar absorptivity of Trp is maximum while those of Phe and Tyr residues are low) and emission wavelength was monitored at 340 nm. (C) Substrate binding affinities (K_D) of *Ec*DHPS Trp mutants for DHPP, *p*ABA and SMX. Substrate-induced fluorescence emission intensity change at 340 nm (error bars represent ±SD) was plotted against substrate concentrations to derive the K_D by GraphPad Prism v5.0. Triplicate readings were averaged, and K_D reported as (±SD). Source data are provided in Source Data.zip.

Fig. S11. Thymidine-auxotrophic phenotype of the unmarked, in-frame *folP* deletion *E. coli* mutant strain.

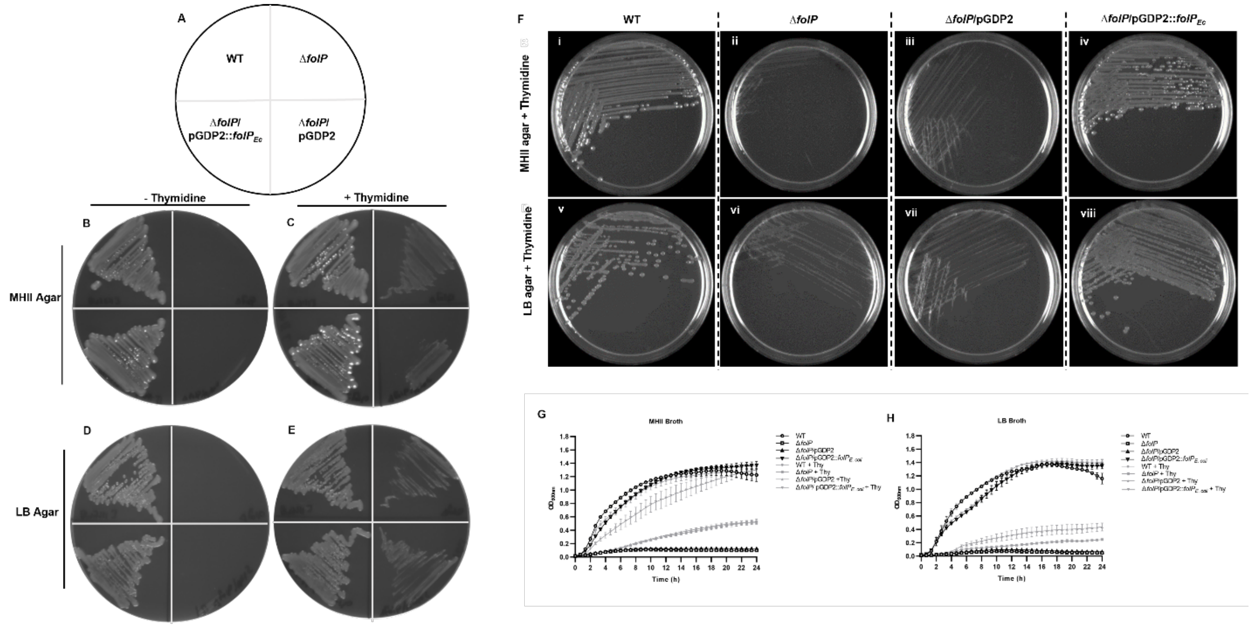

Single colonies of the parent WT *E. coli* BW25113 strain, *E. coli* Δ*folP* strain (Δ*folP*) and its derivatives carrying the empty plasmid, pGDP2, or pGDP2 expressing the WT $folP_{Ec}$ gene were streaked according to the plate map shown in (A) onto either MHII agar without (B; - thymidine) or with 200 µg/mL thymidine (C; +thymidine) or LB agar without (D) or with 200 µg/ml of thymidine (E). (F) MHII agar plates (i-iv) and LB agar plates (v-viii) supplemented with 200 µg/mL thymidine that show the normal phenotype of WT *E. coli* (i and v)) and Δ*folP* strain carrying the plasmid pGDP2 expressing the WT $folP_{Ec}$ gene (iv and viii) and the small colony phenotypes of the Δ*folP* strain(ii and vi) and the Δ*folP* strain carrying the empty plasmid, pGDP2 (iii and vii). (G) Growth curves in MHII broth with and without thymidine (200 µg/mL) for *E. coli* BW25113 (WT), the plasmid-free Δ*folP* parent strain, empty plasmid-carrying Δ*folP* strain (Δ*folP*/pGDP2), or the Δ*folP* strain carrying pGDP2 expressing WT $folP_{Ec}$ gene. (H) Growth curves in LB broth with and without thymidine (200 µg/mL) for *E. coli* BW25113 (WT), the plasmid-free Δ*folP* parent strain, empty plasmid-carrying Δ*folP* strain (Δ*folP*/pGDP2), or the Δ*folP* strain carrying pGDP2 expressing WT $folP_{Ec}$ gene. Data represents the mean ± SEM of three biological independent experiments performed in technical triplicate. ODs at 600 nm were baseline-corrected by subtracting background absorbance at 600 nm of the uninoculated media from the absorbance at 600 nm of the inoculated media. Source data are provided in Source Data.zip.

Fig. S12. Impact of expressing the Sul1, Sul2, or Sul3 enzymes or their variants on the growth of the *E. coli* Δ*folP* strain under thymidine-limited conditions (MHII broth).

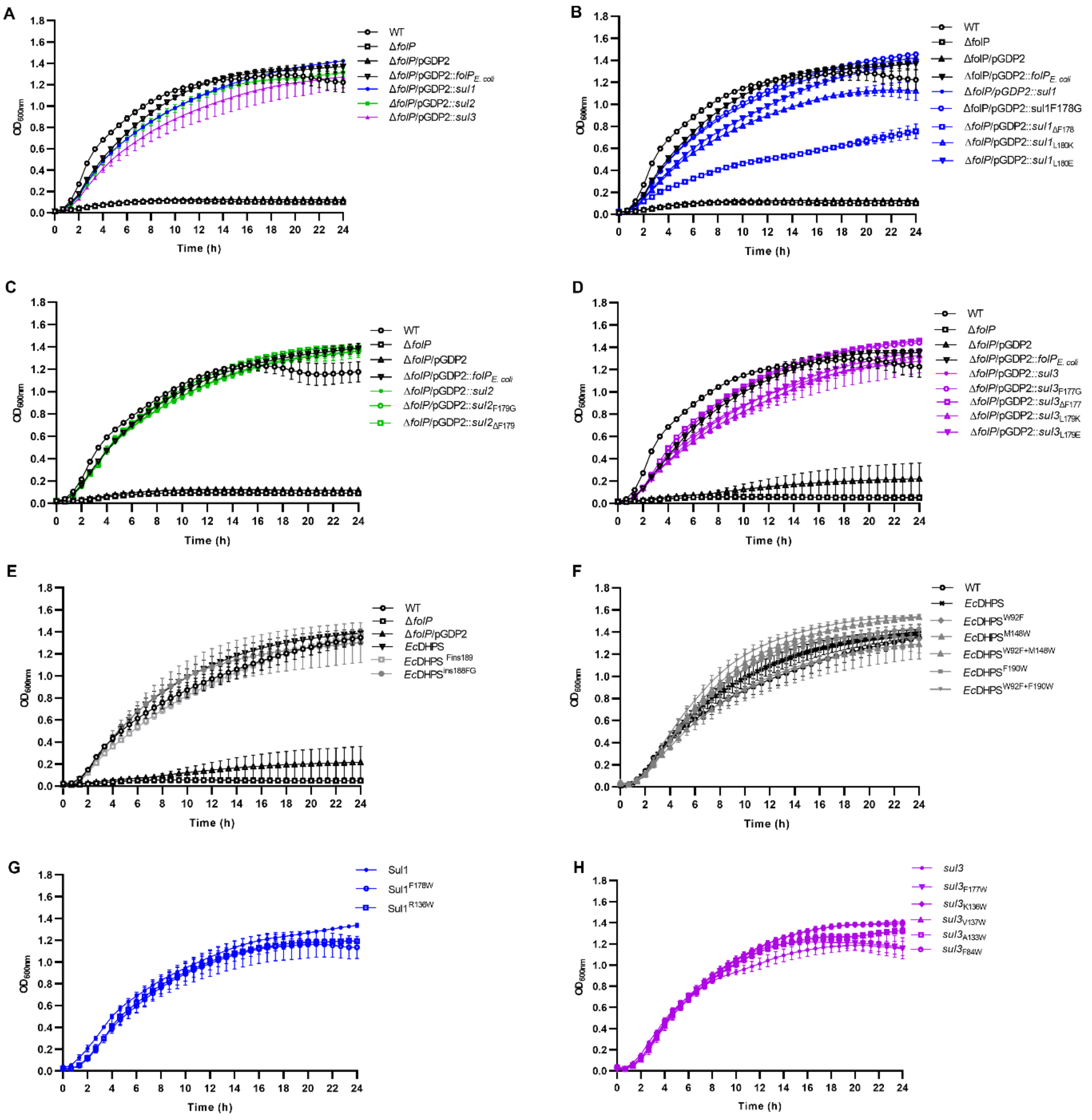

Growth curves for wild-type *E. coli* BW25113 (WT), the plasmid-free Δ*folP* strain (Δ*folP*), empty plasmid-carrying Δ*folP* strain (Δ*folP*/pGDP2), or the Δ*folP* strain carrying pGDP2 expressing either the (A) WT Sul1, Sul2, or Sul3 enzymes, (B) variants of Sul1, (C) variants of Sul2, (D) variants of Sul3, (E) insertion variants of *Ec*DHPS. (F through H) Growth curves of variants of (F) *Ec*DHPS, (G) Sul1 and (H) Sul3 designed for intrinsic tryptophan fluorescence experiments (Trp variants). Data represents the mean ± SEM of three biological independent experiments performed in technical triplicate. ODs at 600 nm were baseline-corrected by subtracting background absorbance at 600 nm of the uninoculated media from the absorbance at 600 nm of the inoculated media. Source data are provided in Source Data.zip.

Fig. S13. Expression of WT and mutant DHPS and Sul proteins in the *E. coli* Δ*folP* strain.

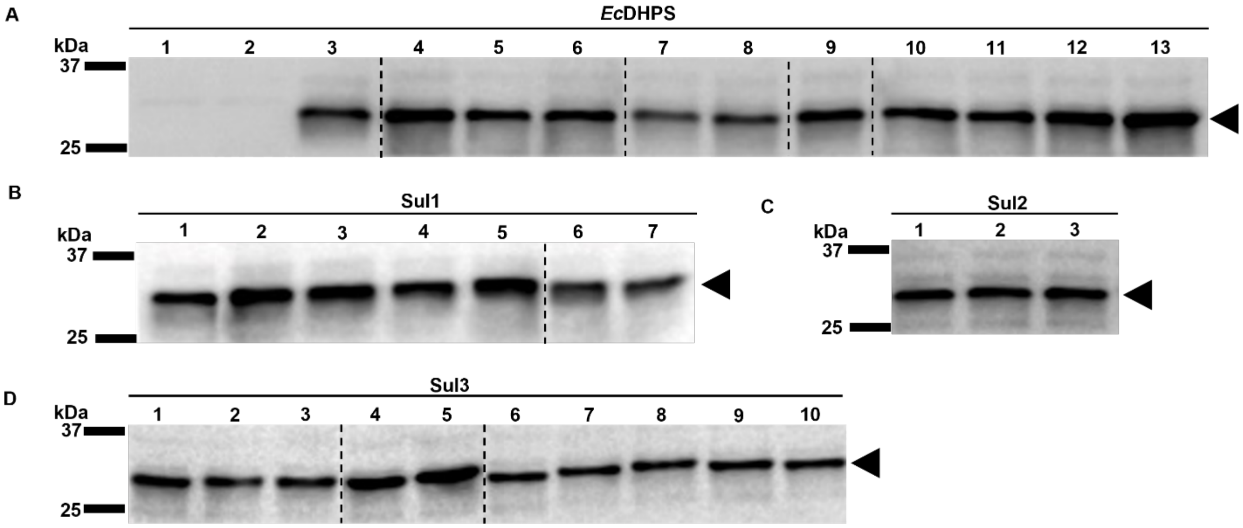

Whole-cell extracts of the *E. coli* Δ*folP* strain expressing plasmid pGDP2-borne, C-terminal FLAG-tagged *Ec*DHPS (A), Sul1 (B), Sul2 (C), and Sul3 (D) enzymes and their mutated derivatives were electrophoretically separated by SDS-PAGE, electroblotted, and developed with monoclonal antibodies directed against the C-terminal flag-tag. (A) Whole-cell extracts *of* the *E. coli* Δ*folP* strain with no plasmid (Lane 1), empty plasmid pGDP2 (Lane 2), or carrying plasmid pGDP2 expressing: WT *Ec*DHPS (Lane 3), *Ec*DHPS^{W92F} (Lane 4), *Ec*DHPS^{F190W} (Lane 5), *Ec*DHPS$^{W92F + F190W}$ (Lane 6), *Ec*DHPS^{M148W} (Lane 7), *Ec*DHPS$^{W92F + M148W}$ (Lane 8), *Ec*DHPS^{insF189} (Lane 9), *Ec*DHPS ^{insFG188} (Lane 10), *Ec*DHPS$^{ins188FG +W92F}$ (Lane 11), *Ec*DHPS$^{ins188FG + F192W}$ (Lane 12), and *Ec*DHPS$^{ins188FG + W92F + F192W}$ (Lane 13). (B) Whole-cell extracts *of* the *E. coli* Δ*folP* strain carrying plasmid pGDP2 expressing: Sul1 (Lane 1), Sul1 F178G (Lane 2), Sul1 ΔF178 (Lane 3), Sul1 L180K (Lane 4); Sul1 L180E (Lane 5); Sul1 F178W (Lane 6), and Sul1 R136W (Lane 7). (C) Whole-cell extracts of the *E. coli* Δ*folP* strain carrying plasmid pGDP2 expressing: Sul2 (Lane 1), Sul2 F178G (Lane 2), and Sul2 ΔF179G. (D) Whole-cell extracts *of* the *E. coli* Δ*folP* strain carrying plasmid pGDP2 expressing: Sul3 (Lane 1), Sul3 F177G (Lane 2), Sul3 ΔF177 (Lane 3), Sul3 L179K (Lane 4), Sul3 L179E (Lane 5), Sul3 F177W (Lane 6), Sul3 K136W (Lane 7), Sul3 V137W (Lane 8), Sul3 A133W (Lane 9), Sul3 F84W (Lane 10). The migration positions of the relevant molecular mass markers are shown on the left of each blot. Vertical dotted lines indicate non-contiguous lanes where the blots were merged. Shown are representative results from two biological replicates. Source data are provided in Source Data.zip.

Fig. S14. AlphaFold2 model of *Nm*DHPS^{insSG}.

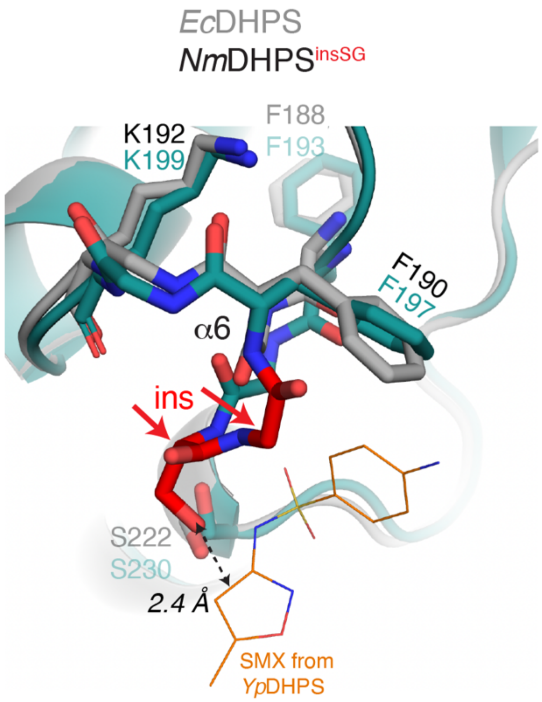

Model of the *Nm*DHPS enzyme (Uniprot ID Q51161) generated by AlphaFold2. Shown is the α6 region with insertion SG (*Nm*DHPS^{insSG}) that confers sulfonamide resistance (from Fermer *et al*, *J Bacteriol.* 1995 Aug;177(16)-4669-75) overlaid with the structure of *Ec*DHPS (PDB 1AJZ, Achari A *et al*, *Nat. Struct. Biol.* 1997 4:490-497) and SMX from the structure of *Yp*DHPS (PDB 3TZF, Yun MK *et al*, *Science* 2012 335:1110-1114). The *Nm*DHPS insertion Ser-Gly amino acids are coloured red and labeled. Source data are provided in Source Data.zip.

Fig. S15. Molecular dynamics simulations of Sul2 and *Ec*DHPS.

A

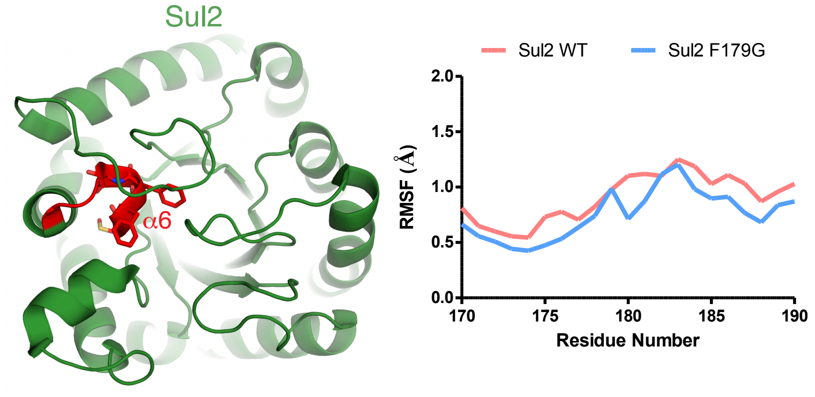

Residue	RMSF Sul2 WT	RMSF Sul2 F179G	ΔRMSF
G176	0.78	0.53	0.24
M177	0.71	0.64	0.07
G178	0.82	0.74	0.08
F179	0.98	0.99	-0.01
F180	1.10	0.71	0.39
L181	1.12	0.88	0.24
G182	1.10	1.12	-0.02
A183	1.25	1.20	0.05
A184	1.19	0.98	0.21

B

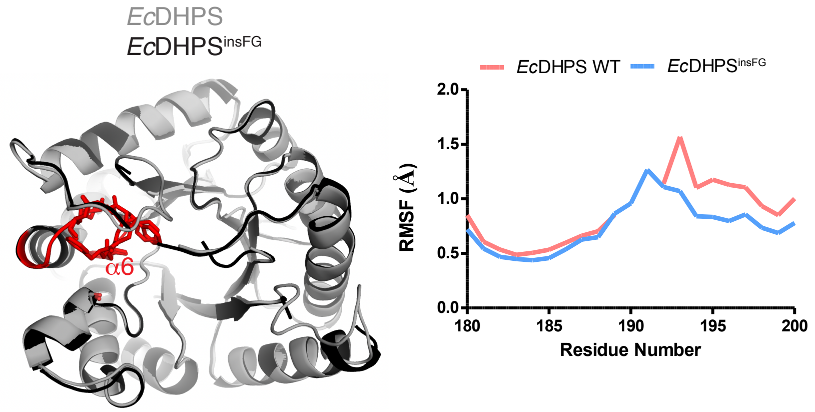

Residue	RMSF *Ec*DHPS WT	RMSF *Ec*DHPS^{insFG}	ΔRMSF
G187	0.66	0.63	0.03
F188	0.70	0.65	0.05
G189	0.85	0.86	-0.01
F190	-	0.96	N/A
G191	-	1.27	N/A
K192	1.14	1.11	0.03
N193	1.57	1.07	0.50
L194	1.10	0.84	0.26
S195	1.18	0.83	0.34

A) Left = structure of Sul2 with α6 region highlighted in red (focus of study). Middle = plot of root-mean-square-fluctuation (RMSF) after 200 ns MD simulation for Sul2 WT (red plot) and Sul2 F179G (blue plot). Right = RMSF per residue in α6 region and change in RMSF between WT and F179G simulations. B) Left = structure of *Ec*DHPS WT (grey) and *Ec*DHPS^{insFG} (black) with α6 region highlighted in red. Middle = plot of RMSF after 200 ns MD simulation for *Ec*DHPS WT (red plot) and *Ec*DHPS^{insFG} (blue plot). Right = RMSF per residue in α6 region and change in RMSF between WT and insFG simulations. Source data are provided in Source Data.zip.

SUPPLEMENTARY TABLES

Table S1. X-ray crystallographic statistics.

	**Sul1•6-HMP**	**Sul2 apoenzyme**	**Sul2•7,8-DHP•Mg^{2+}•PP_i**
**PDB code**	7S2I	7S2J	7S2K
**Data collection**			
Space group	$P4_132$	$P2_1$	$P2_1$
Cell dimensions			
a, b, c (Å)	186.6, 186.6, 186.6	39.8, 143.3, 85.9	45.7, 74.2, 67.9
α, β, γ, (°)	90, 90, 90	90, 90.5, 90	90, 96.1, 90
Resolution, Å	30.00 – 2.32	50.00 – 1.85	30.0 – 1.74
R_{merge}[a]	0.119 (2.135)[*]	0.105 (0.764)	0.071 (0.396)
R_{pim}[b]	0.034 (0.520)	0.043 (0.328)	0.034 (0.200)
$CC_{1/2}$[*]	0.520	0.912	0.994
$I / \sigma (I)$	24.5 (1.04)	19.3 (2.07)	20.9 (2.2)
Completeness, %	100 (100)	99.4 (99.3)	98.0 (97.4)
Redundancy	13.4 (12.6)	6.6 (6.2)	4.6 (4.3)
**Refinement**			
Resolution, Å	29.14 – 2.32	42.94 – 1.85	28.76 – 1.74
No. unique reflections: working, test	48269, 2000	81545, 3896	45534, 1999
R_{work}/R_{free}[c]	17.7/19.5 (28.1/29.7)	16.2/20.7 (25.7/34.4)	18.0/21.3 (24.0/31.4)
No. atoms			
Protein	4008	7963	3911
Ligands	28	N/A	66
Solvent	61	89	7
Water	289	1168	394
B-factors			
Protein	72.3	35.9	30.5
Ligands	53.8	N/A	28.3
Solvent	116.5	52.6	39.7
Water	75.7	41.8	36.9
R.m.s. deviations			
Bond lengths, Å	0.005	0.006	0.006
Bond angles, °	0.763	0.895	1.012

	**Sul3 apoenzyme**	**Sul3•6-HMP**	**Sul3•DHP^{+}•*p*ABA•Mg^{2+}•PP_i**	***Ec*DHPS^{insFG}·6-HMP**
**PDB code**	7S2L	7S2M	8SCD	7TQ1
**Data collection**				
Space group	$P6_222$	$P3_112$	$P3_22_1$	I222
Unit cell<br>*a*, *b*, *c* (Å)<br>α, β, γ, (°)	 123.8, 123.8, 434.4<br>90, 90, 120	 50.1, 50.1, 583.0<br>90, 90, 120	 123.8, 123.8, 728<br>90, 90, 120	 77.9, 84.6, 175.5<br>90, 90, 90
Resolution, Å[#]	30.00 – 2.79	30.00 – 2.36	50.0 – 2.07	50.0 – 2.75
R_{merge}[a]<br>R_{pim}[b]	0.153 (2.124)[*]<br>0.060 (0.815)	0.093 (0.738)<br>0.050 (0.457)	0.137 (3.178)<br>0.032 (0.795)	 0.189 (0.945)<br>0.081 (0.435)
$CC_{1/2}$[*]	0.994 (0.587	0.749	0.504	0.764
$I / \sigma (I)$	16.38 (1.06)	25.39 (1.0)	20.58 (1.13)	10.05 (1.04)
Completeness, %	100 (100)	98.8 (86.7)	100 (100)	95.4 (82.2)
Redundancy	27.2 (19.7)	6.4 (3.4)	19.5 (16.9)	6.6 (5.4)
**Refinement**				
Resolution, Å	30.00 – 2.79	30.04 – 2.42	47.0 – 2.00	46.77 – 2.75
No. unique reflections: working, test	49650, 3687	26784, 1335	36295, 3177	2305, 119
R_{work}/ R-$_{free}$[c]	22.8/27.0 (40.4/40.4)	25.0/30.0 (33.7/34.4)	16.5/18.8 (34.8/28.6)	23.6/28.8 (33.1/42.4)
No. atoms,<br>Protein<br>Ligands<br>Solvent<br>Water	 7965<br>N/A<br>91<br>128	 5900<br>42<br>N/A<br>352	 2044<br>33<br>17<br>446	 4124<br>28<br>N/A<br>52
B-factors<br>Protein<br>Ligand<br>Solvent<br>Water	 76.0<br>N/A<br>97.3<br>63.3	 43.1<br>23.3<br>N/A<br>36.1	 36.6<br>26.9<br>73.3<br>53.2	 77.1<br>75.3<br>N/A<br>61.5
R.m.s. deviations<br>Bond lengths, Å<br>Bond angles, °	 0.005<br>0.771	 0.002<br>0.546	 0.012<br>0.491	 0.004<br>0.646

[#]one crystal was used for each structure.
[*]Values in parentheses and $CC_{1/2}$ values are for highest resolution shells.
N/A = not applicable.

Table S2. Sulfonamide susceptibility of the *E. coli* Δ*folP* deletion expressing the WT or mutated Sul enzymes.[a]

				MIC (µg/mL)[b]												MIC (µg/mL)[g]
Strain	Plasmid	DHPS[c]	Growth on MHII agar[d]	SMX	SDZ	SOZ	SPY	STZ	SMRZ	SMZ	SAA	SMT[e]	SAD	SQX[f]	SDT	SXT (SMX-TMP)
WT	None	*Ec*DHPS	G	16	32	32	64	16	32	256	2048	128	512	512	256	0.094
Δ*folP*	None	None	NG	NG	NG	NG	NG	NG	NG	NG	NG	NG	NG	NG	NG	NG
Δ*folP*	pGDP2	None	NG	NG	NG	NG	NG	NG	NG	NG	NG	NG	NG	NG	NG	NG
Δ*folP*	pGDP2	*Ec*DHPS	G	16	32	32	64	16	32	256	2048	128	512	512	256	0.064
Δ*folP*	pGDP2	Sul1	G	2048	4096	2048	4096	4096	4096	4096	8192	>4096	2048	>1024	8192	0.19
Δ*folP*	pGDP2	Sul1^{F178G}	G	16	32	64	64	64	32	128	4096	64	256	64	256	0.064
Δ*folP*	pGDP2	Sul1$^{F\Delta178}$	NG	NG	NG	NG	NG	NG	NG	NG	NG	NG	NG	NG	NG	NG
Δ*folP*	pGDP2	Sul1^{L180K}	G	2048	1024	2048	256	256	1024	2048	4096	>4096	2048	>1024	2048	0.19
Δ*folP*	pGDP2	Sul1^{L180E}	G	64	64	2048	64	32	64	256	1024	256	1024	256	512	0.023
Δ*folP*	pGDP2	Sul2	G	2048	4096	2048	4096	4096	4096	4096	8192	>4096	2048	>1024	8192	0.19
Δ*folP*	pGDP2	Sul2^{F179G}	G	32	64	128	128	128	128	512	4096	512	256	512	512	0.064
Δ*folP*	pGDP2	Sul2$^{\Delta F179}$	G	4	8	4	4	2	32	32	128	16	32	32	32	0.016
Δ*folP*	pGDP2	Sul3	G	2048	4096	2048	4096	4096	4096	4096	8192	>4096	2048	>1024	8192	0.19
Δ*folP*	pGDP2	Sul3^{F177G}	G	16	32	128	128	16	64	512	4096	512	512	512	256	0.064
Δ*folP*	pGDP2	Sul3$^{\Delta F177}$	G	2	8	16	16	2	16	32	256	32	32	16	8	0.023
Δ*folP*	pGDP2	Sul3^{L179K}	G	2048	4096	2048	4096	4096	4096	4096	8192	>4096	2048	>1024	8192	0.25
Δ*folP*	pGDP2	Sul3^{L179E}	G	64	128	256	64	16	128	1024	1024	>4096	2048	>1024	128	0.094

Sensitive

Resistant

[a] The sulfa susceptibility of the WT *E. coli* BW25113 (WT) strain and the *E. coli ΔfolP* strain carrying the indicated plasmids expressing wild-type *Ec*DHPS or Sul1, Sul2, Sul3 or Sul1, Sul2, and Sul3 variants with the indicated amino acid substitutions or deletions is reported. Results for the WT *E. coli* BW25113 strain is provided for comparison purposes. Results for the plasmid-free and empty plasmid-carrying (pGDP2) *E. coli folP* deletion are provided to confirm the absence of thymidine in the MHII agar media. For all strains and drugs tested, 3 biological replicates were performed.

[b] SMX, sulfamethoxazole; SDZ, Sulfadiazine; SOZ, sulfisoxazole; SPY, sulfapyridine; STZ, sulfathiazole; SMRZ, sulfamerazine; SMZ, sulfamethazine; SAA, sulfanilamide; SMT, sulfameter; SAD, sulfacetamide; SQX, sulfaquinoxaline; and SDT, sulfadimethoxine. Susceptibility testing for these agents was conducted using the agar dilution method and MHII agar.

[c] DHPS status of the indicated strains; *Ec*DHPS, *E. coli* WT DHPS enzyme.

[d] NG, No growth; or G, growth on MHII agar.

[e] For SMT MIC values of >4096 µg/mL indicate that the MIC is greater than 4096 µg/mL. SMT concentrations greater than 4096 µg/mL were not tested because of the drug's limited solubility in MHII agar.

[f] For SQX, MIC values of >1024 µg/mL indicate that the MIC is greater than 1024 µg/mL. SQX concentrations greater than 1024 µg/mL were not tested because of the drug's limited solubility in MHII agar.

[g] SXT, sulfamethoxazole (SMX)-trimethoprim (TMP) (1:19), values indicate TMP concentration. MICs for SXT were determined using the E-test method and MHII agar. Source data are provided in Source Data.zip.

Table S3. Sulfa susceptibility of the *E. coli* Δ*folP* deletion expressing the *Ec*DHPS, Sul1 or Sul3 tryptophan substitution mutant derivatives.[a]

Strain	Plasmid	DHPS[c]	Growth on MHII agar[d]	MIC (µg/mL)[b] SMX	SDZ	SOZ
WT	None	*Ec*DHPS	G	16	32	32
Δ*folP*	pGDP2	*Ec*DHPS	G	16	32	32
Δ*folP*	pGDP2	*Ec*DHPS$^{\text{W92F}}$	G	32	32	64
Δ*folP*	pGDP2	*Ec*DHPS$^{\text{F190W}}$	G	8	16	16
Δ*folP*	pGDP2	*Ec*DHPS$^{\text{W92F F190W}}$	G	16	16	16
Δ*folP*	pGDP2	*Ec*DHPS$^{\text{M148W}}$	G	8	16	16
Δ*folP*	pGDP2	*Ec*DHPS$^{\text{W92F M148W}}$	G	4	16	8
Δ*folP*	pGDP2	*Ec*DHPS$^{\text{ins188FG}}$	G	32	128	64
Δ*folP*	pGDP2	*Ec*DHPS$^{\text{ins188FG W92F}}$	G	64	256	128
Δ*folP*	pGDP2	*Ec*DHPS$^{\text{ins188FG F192W}}$	G	16	64	64
Δ*folP*	pGDP2	*Ec*DHPS$^{\text{ins188FG W92F F192W}}$	G	32	64	64
Δ*folP*	pGDP2	Sul1	G	2048	4096	2048
Δ*folP*	pGDP2	Sul1$^{\text{F178W}}$	G	2048	4096	4096
Δ*folP*	pGDP2	Sul1$^{\text{R136W}}$	G	2048	4096	4096
Δ*folP*	pGDP2	Sul3	G	2048	4096	2048
Δ*folP*	pGDP2	Sul3$^{\text{F177W}}$	G	2048	4096	4096
Δ*folP*	pGDP2	Sul3$^{\text{K136W}}$	G	2048	4096	4096
Δ*folP*	pGDP2	Sul3$^{\text{V137W}}$	G	2048	4096	4096
Δ*folP*	pGDP2	Sul3$^{\text{A33W}}$	G	2048	4096	4096
Δ*folP*	pGDP2	Sul3$^{\text{F84W}}$	G	2048	4096	4096

Sensitive

Resistant

[a] The sulfa susceptibility of the WT *E. coli* BW25113 (WT) strain and the *E. coli ΔfolP* strain carrying the indicated plasmids expressing wild-type *Ec*DHPS, *Ec*DHPS insFG188, Sul1, Sul3 or their tryptophan substitution mutant derivatives with the indicated amino acid is reported. Results for the *E. coli* BW25113 strain (WT) is provided for comparison purposes. For all strains and drugs tested, 3 biological replicates were performed using the agar dilution method and MHII agar.

[b] SMX, sulfamethoxazole; SDZ. Sulfadiazine; SOZ, sulfisoxazole.

[c] DHPS status of the indicated strains ; *Ec*DHPS, *E. coli* WT DHPS enzyme

[d] NG, No growth; or G, growth on MHII agar.

Source data are provided in Source Data.zip.

Table S4. Sulfa susceptibility[a] of the sulfamethoxazole-resistant DHPS mutants, *Ec*DHPS$^{\text{ins188FG}}$ of *E. coli* BW25113 selected following a 7-day sulfanilamide exposure and susceptibility of the Δ*folP* strain expressing plasmid-borne *Ec*DHPS$^{\text{ins188FG}}$.

Sensitive

			MIC (µg/mL)[b]												MIC (µg/mL)[e]
Strain	Plasmid	DHPS	SMX	SDZ	SOZ	SPY	STZ	SMRZ	SMZ	SAA	SMT[c]	SAD	SQX[d]	SDT	SXT (SMX-TMP)[e]
WT	None	*Ec*DHPS	16	32	32	64	16	32	256	2048	128	512	512	256	0.094
Δ*folP*	pGDP2	*Ec*DHPS	16	32	32	64	16	32	256	2048	128	512	512	256	0.064
SMX^{R}-1.4	None	*Ec*DHPS$^{\text{ins188FG}}$	256	512	256	ND[f]	ND	ND	ND	4096	ND	ND	ND	ND	ND
SMX^{R}-3.4	None	*Ec*DHPS$^{\text{ins188FG}}$	256	512	512	ND	ND	ND	ND	4096	ND	ND	ND	ND	ND
SMX^{R}-4.4	None	*Ec*DHPS$^{\text{ins188FG}}$	256	512	512	ND	ND	ND	ND	4096	ND	ND	ND	ND	ND
Δ*folP*	pGDP2	*Ec*DHPS$^{\text{ins188FG}}$	32	128	64	256	64	256	512	4096	1024	1024	>1024	512	0.125
Δ*folP*	pGDP2	*Ec*DHPS$^{\text{Fins189}}$	8	16	16	64	8	8	64	256	64	128	256	64	0.047

Resistant

[a] Wild-type *E. coli* BW25113 was exposed to sulfanilamide (half the MIC; 1024 µg/mL) over 7 days and mutants resistant to 256 µg/mL of sulfamethoxazole (SMX) were selected. Results for the *E. coli* BW25113 strain (WT) and for the three SMX-resistant mutants (SMX^{R}) harboring an FG insertion at position 188 of the WT *Ec*DHPS coding region are reported. The antimicrobial susceptibility of *E. coli* Δ*folP* strain carrying the plasmid pGDP2 expressing (WT) *Ec*DHPS or *Ec*DHPS derivatives with the indicated amino acid insertions are also reported. For all strains and drugs tested, 3 biological replicates were performed using the agar dilution method and MHII agar.

[b] SMX, sulfamethoxazole; SDZ, Sulfadiazine; SOZ, sulfisoxazole; SPY, sulfapyridine; STZ, sulfathiazole; SMRZ, sulfamerazine; SMZ, sulfamethazine; SAA, sulfanilamide; SMT, sulfameter; SAD, sulfacetamide; SQX, sulfaquinoxaline; and SDT, sulfadimethoxine.

[c] For SMT MIC values of >4096 µg/mL indicate that the MIC is greater than 4096 µg/mL. SMT concentrations greater than 4096 µg/mL were not tested because of the drug's limited solubility in MHII agar.

[d] For SQX, MIC values of >1024 µg/mL indicate that the MIC is greater than 1024 µg/mL. SQX concentrations greater than 1024 µg/mL were not tested because of the drug's limited solubility in MHII agar.

[e] SXT, sulfamethoxazole (SMX)-trimethoprim (TMP) (1:19), values indicate TMP concentration. MICs for SXT were determined using the E-test method and MHII agar.

[f] ND, not determined.

Source data are provided in Source Data.zip.

Table S5. Bacterial strains and plasmids used in this study.

Strain	Relevant Genotype	Source
E. coli		
DH5α	*φ80ΔlacZΔM15 endA1 recA1 hsdR17(rK -mK+) supE44 thi-1 gyrA96 relA1 F- Δ(lacZYA-argF) U169*	Ausubel FM, Brent R, Kingston RE, Moore DD, Seidman JG, et al. (1992) Short protocols in molecular biology, 2nd ed. New York: John Wiley & Sons, Inc.
BW25113	*E. coli* K-12 BW25113 wild type: Δ(*araD-araB*)*567* Δ*lacZ4787*(::*rrnB-3*) *rph-1* Δ(*rhaD-rhaB*)*568 hsdR514*	Keio Collection
Δ*folP*	BW25113 carrying an unmarked, in-frame *folP* gene deletion	This study
BL21(DE3) Gold	*E. coli B F- dcm+ The ompT hsdS(r_B-m_B-) gal λ (DE3) endA Tet^r*	Agilent
SMX^R-14.1	Sulfa-resistant BW25113 derivative carrying a $folP_{T62K}$ mutation that was selected following a 7-day exposure to 1024 µg/mL of sulfanilamide	This study
SMX^R-20.1	Sulfa-resistant BW25113 derivative carrying a $folP_{T62A}$ mutation that was selected following a 7-day exposure to 1024 µg/mL of sulfanilamide	This study
SMX^R-22.1	Sulfa-resistant BW25113 derivative carrying a $folP_{T62K}$ mutation that was selected following a 7-day exposure to 1024 µg/mL of sulfanilamide	This study
SMX^R-24.1	Sulfa-resistant BW25113 derivative carrying a $folP_{T62A}$ mutation that was selected following a 7-day exposure to 1024 µg/mL of sulfanilamide	This study
SMX^R-26.1	Sulfa-resistant BW25113 derivative carrying a $folP_{T62A}$ mutation that was selected following a 7-day exposure to 1024 µg/mL of sulfanilamide	This study
SMX^R-12.2	Sulfa-resistant BW25113 derivative carrying a $folP_{T62A}$ mutation that was selected following a 7-day exposure to 1024 µg/mL of sulfanilamide	This study
SMX^R-42.2	Sulfa-resistant BW25113 derivative carrying a $folP_{T62A}$ mutation that was selected following a 7-day exposure to 1024 µg/mL of sulfanilamide	This study
SMX^R-46.2	Sulfa-resistant BW25113 derivative carrying a $folP_{T62A}$ mutation that was selected following a 7-day exposure to 1024 µg/mL of sulfanilamide	This study
SMX^R-48.2	Sulfa-resistant BW25113 derivative carrying a $folP_{T62A}$ mutation that was selected following a 7-day exposure to 1024 µg/mL of sulfanilamide	This study
SMX^R-60.2	Sulfa-resistant BW25113 derivative carrying a $folP_{T62A}$ mutation that was selected following a 7-day exposure to 1024 µg/mL of sulfanilamide	This study
SMX^R-12.3	Sulfa-resistant BW25113 derivative carrying a $folP_{T62A}$ mutation that was selected following a 7-day exposure to 1024 µg/mL of sulfanilamide	This study
SMX^R-16.3	Sulfa-resistant BW25113 derivative carrying a $folP_{T62A}$ mutation that was selected following a 7-day exposure to 1024 µg/mL of sulfanilamide	This study
SMX^R-18.3	Sulfa-resistant BW25113 derivative carrying a $folP_{T62A}$ mutation that was selected following a 7-day exposure to 1024 µg/mL of sulfanilamide	This study
SMX^R-20.3	Sulfa-resistant BW25113 derivative carrying a $folP_{T62A}$ mutation that was selected following a 7-day exposure to 1024 µg/mL of sulfanilamide	This study

SMX^R-22.3	Sulfa-resistant BW25113 derivative carrying a *folP*$_{T62A}$ mutation that was selected following a 7-day exposure to 1024 µg/mL of sulfanilamide	This study
SMX^R-22.3	Sulfa-resistant BW25113 derivative carrying a *folP*$_{T62A}$ mutation that was selected following a 7-day exposure to 1024 µg/mL of sulfanilamide	This study
SMX^R-22.3	Sulfa-resistant BW25113 derivative carrying a *folP*$_{T62A}$ mutation that was selected following a 7-day exposure to 1024 µg/mL of sulfanilamide	This study
SMX^R-1.4	Sulfa-resistant BW25113 derivative carrying a *folP* $_{Gly187\_Phe188insPheGly}$ mutation that was selected following a 7-day exposure to 1024 µg/mL of sulfanilamide	This study
SMX^R-2.4	Sulfa-resistant BW25113 derivative with no mutation in *folP* that was selected following a 7-day exposure to 1024 µg/mL of sulfanilamide	This study
SMX^R-3.4	Sulfa-resistant BW25113 derivative carrying a *folP* $_{Gly187\_Phe188insPheGly}$ mutation that was selected following a 7-day exposure to 1024 µg/mL of sulfanilamide	This study
SMX^R-4.4	Sulfa-resistant BW25113 derivative carrying a *folP* $_{Gly187\_Phe188insPheGly}$ mutation that was selected following a 7-day exposure to 1024 µg/mL of sulfanilamide	This study
SMX^R-5.4	Sulfa-resistant BW25113 derivative with no mutation in *folP* that was selected following a 7-day exposure to 1024 µg/mL of sulfanilamide	This study
**Plasmids**		
pUC19	*E. coli* gene expression vector, Ap^R	PMID:6323249
pMJF200	pUC19:: Δ*folP* upstream fragment	This study
pMJF201	pUC19:: Δ*folP* downstream fragment	This study
pMJF202	pUC19:: Δ*folP*	This study
pKOV	*repA101*(Ts) *sacB* Cm^r ; Gene replacement vector	PMID:9335267
pMJF203	pKOV::Δ*folP*	This study
pMCSG53	N-terminal His$_6$-tag expression vector; Ap^R	PMID:24057978
pNIC-CH	C-terminal His$_6$-tag expression vector; Km^R	
pGDP2	*E. coli* gene expression vector; Km^R	PMID:28017602
pMJF204	pGDP2::*sul1*$_{WT}$-FLAG	This study
pMJF205	pGDP2::*sul1*$_{F178G}$-FLAG	This study
pMJF206	pGDP2::*sul1*$_{\Delta 178}$-FLAG	This study
pMJF207	pGDP2::*sul1*$_{L180K}$-FLAG	This study
pMJF208	pGDP2::*sul1*$_{L180E}$-FLAG	This study
pMJF209	pGDP2::*sul1*$_{F178W}$-FLAG	This study

pMJF210	pGDP2::*sul1*$_{R136W}$-FLAG	This study
pMJF211	pGDP2::*sul2*$_{WT}$-FLAG	This study
pMJF212	pGDP2::*sul2*$_{F179G}$-FLAG	This study
pMJF213	pGDP2::*sul2*$_{\Delta F179}$-FLAG	This study
pMJF214	pGDP2::*sul3*$_{WT}$-FLAG	This study
pMJF215	pGDP2::*sul3*$_{F177G}$-FLAG	This study
pMJF216	pGDP2::*sul3*$_{\Delta F177}$-FLAG	This study
pMJF217	pGDP2::*sul3*$_{L179K}$-FLAG	This study
pMJF218	pGDP2::*sul3*$_{L179E}$-FLAG	This study
pMJF219	pGDP2::*sul3*$_{F177W}$-FLAG	This study
pMJF220	pGDP2::*sul3*$_{K136W}$-FLAG	This study
pMJF213	pGDP2::*sul3*$_{V137W}$-FLAG	This study
pMJF221	pGDP2::*sul3*$_{A33W}$-FLAG	This study
pMJF222	pGDP2::*sul3*$_{F84W}$-FLAG	This study
pMJF223	pGDP2::*folP*$_{Ec}$-FLAG	This study
pMJF224	pGDP2::*folP*$_{Gly187\_Phe188insPheGly}$ –FLAG	This study
pMJF225	pGDP2::*folP*$_{Gly189\_Phe190insPhe}$ –FLAG	This study
pMJF226	pGDP2::*folP*$_{W92F}$ –FLAG	This study
pMJF227	pGDP2::*folP*$_{F190W}$ –FLAG	This study
pMJF228	pGDP2::*folP*$_{W92F+F190W}$ –FLAG	This study
pMJF229	pGDP2::*folP*$_{F148W}$ –FLAG	This study
pMJF230	pGDP2::*folP*$_{W92F+M148W}$ –FLAG	This study
pMJF231	pGDP2::*folP*$_{W92F+M148W}$ –FLAG	This study
pMJF232	pGDP2::*folP*$_{W92F+Gly189\_Phe190insPhe}$ –FLAG	This study
pMJF233	pGDP2::*folP*$_{W92F+Gly189\_Phe190insPhe +F192W}$ –FLAG	This study
pMJF234	pGDP2::*folP*$_{W92F+Gly189\_Phe190insPhe+F192W}$ –FLAG	This study

Note: Ap^R ampicillin resistant; Cm^R chloramphenicol resistant; Km^R, kanamycin resistant; SMX^R, sulfamethoxazole resistant; WT, wild type; FLAG, FLAG-tag.

Table S6. Primers used in this study.

Primer Name	Sequence (5'-3')
Δ*folP* Up For	GACTAAGCTTCAGGTTGTGGTCGGCTTGCC<br>HindIII
Δ*folP* Up Rev	GACTTCTAGAGGCAAAGAGTTTCATGATGTTATCCCTGG<br>XbaI
Δ*folP* Dn For	GACTTCTAGAGTGGTGGAAGCCACTCTGTCTGCA<br>XbaI
Δ*folP* Dn Rev	GACTGGATCCAGCCAGTTATCTAACGCTTT<br>BamHI
Δ*folP* For NotI	GACTGCGGCCGCCAGGTTGTGGTCGGCTTGCC<br>NotI
folP For	CGACGCACCGCAGATTGATGACCTG
folP Rev	CCAGTGCTGACTCCAGCATATAGCC
folP$_{\text{ins188FG}}$ For	GACTTCTAGATTAACTTTAAGAAGGAGATATACATGAAACTCTTTGCC<br>XbaI<br>CAGGGTAC
folP$_{\text{ins188FG}}$ Rev	GACTAAGCTT**TTA***CTTGTCGTCATCGTCTTTGTAGTC*CTCATAGCGTT<br>HindIII<br>TGTTTTCCTTT

Note: Underlined sequence demarcate restriction enzyme cut sites; bolded sequence codes for a stop codon; italicized sequence codes for the FLAG-tag.
