# Supplementary material for: Molecular mechanism of plasmid-borne resistance to sulfonamide antibiotics
Source: Nat Commun. 2023 Jul 7;14:4031. doi: 10.1038/s41467-023-39778-7 (PMC10328974; doi:10.1038/s41467-023-39778-7)
Supplement: Supplementary file 3 — Reporting Summary [file 41467_2023_39778_MOESM3_ESM.pdf]

nature portfolio

Corresponding author(s): Peter Stogios and Alexei Savchenko

Last updated by author(s): Jun 7, 2023

Reporting Summary

Nature Portfolio wishes to improve the reproducibility of the work that we publish. This form provides structure for consistency and transparency in reporting. For further information on Nature Portfolio policies, see our Editorial Policies and the Editorial Policy Checklist.

Statistics

For all statistical analyses, confirm that the following items are present in the figure legend, table legend, main text, or Methods section.

n/a	Confirmed	
☐	☒	The exact sample size (*n*) for each experimental group/condition, given as a discrete number and unit of measurement
☐	☒	A statement on whether measurements were taken from distinct samples or whether the same sample was measured repeatedly
☒	☐	The statistical test(s) used AND whether they are one- or two-sided<br>*Only common tests should be described solely by name; describe more complex techniques in the Methods section.*
☒	☐	A description of all covariates tested
☒	☐	A description of any assumptions or corrections, such as tests of normality and adjustment for multiple comparisons
☐	☒	A full description of the statistical parameters including central tendency (e.g. means) or other basic estimates (e.g. regression coefficient) AND variation (e.g. standard deviation) or associated estimates of uncertainty (e.g. confidence intervals)
☒	☐	For null hypothesis testing, the test statistic (e.g. *F*, *t*, *r*) with confidence intervals, effect sizes, degrees of freedom and *P* value noted<br>*Give P values as exact values whenever suitable.*
☒	☐	For Bayesian analysis, information on the choice of priors and Markov chain Monte Carlo settings
☒	☐	For hierarchical and complex designs, identification of the appropriate level for tests and full reporting of outcomes
☒	☐	Estimates of effect sizes (e.g. Cohen's *d*, Pearson's *r*), indicating how they were calculated

Our web collection on statistics for biologists contains articles on many of the points above.

Software and code

Policy information about availability of computer code

Data collection	GraphPad Prism v5.0<br>HKL3000 v. 718.3<br>PHENIX v. 1.20.4459<br>Coot v. 0.8.9.1<br>PyMOL v. 2.4.0<br>Thermo Xcalibur Qual Browser v.3.1<br>GROMACS package version 2020.2<br>SKANIT Software CF v.7.0<br>DNR Bio-Imaging Systems MicroChemi v.4.2

March 2021

Data analysis	GraphPad Prism v5.0<br>HKL3000 v. 718.3<br>PHENIX v. 1.20.4459<br>Coot v. 0.8.9.1<br>PyMOL v. 2.4.0<br>Thermo Xcalibur Qual Browser v.3.1<br>GROMACS package version 2020.2<br>SKANIT Software CF v.7.0<br>DNR Bio-Imaging Systems MicroChemi v.4.2

For manuscripts utilizing custom algorithms or software that are central to the research but not yet described in published literature, software must be made available to editors and reviewers. We strongly encourage code deposition in a community repository (e.g. GitHub). See the Nature Portfolio guidelines for submitting code & software for further information.

Data

Policy information about availability of data

All manuscripts must include a data availability statement. This statement should provide the following information, where applicable:

- Accession codes, unique identifiers, or web links for publicly available datasets
- A description of any restrictions on data availability
- For clinical datasets or third party data, please ensure that the statement adheres to our policy

Atomic coordinates have been deposited in the Protein Data Bank with accession codes 7S2I, 7S2J, 7S2K, 7S2L, 7S2M, 7TQ1 and 8SCD. Start and final configurations for MD simulations are provided in the Supplementary Data 1. The enzyme kinetics, ITF, MIC and growth curve, mass spectrometry and Western blot data generated in this study are provided in the Supplementary Data 2. Databases used in the study: Protein Data Bank (accession code 1AJ0) and NCBI Genbank.

Human research participants

Policy information about studies involving human research participants and Sex and Gender in Research.

Reporting on sex and gender	N/A
Population characteristics	N/A
Recruitment	N/A
Ethics oversight	N/A

Note that full information on the approval of the study protocol must also be provided in the manuscript.

Field-specific reporting

Please select the one below that is the best fit for your research. If you are not sure, read the appropriate sections before making your selection.

[x] Life sciences [ ] Behavioural & social sciences [ ] Ecological, evolutionary & environmental sciences

For a reference copy of the document with all sections, see nature.com/documents/nr-reporting-summary-flat.pdf

Life sciences study design

All studies must disclose on these points even when the disclosure is negative.

Sample size	Triplicate E. coli strains following the Clinical and Laboratory Standards Institute (CLSI) protocol for the microbroth dilution method; this sample size was chosen as is typical in the literature for minimum inhibitory concentration (MIC) calculations. Biological triplicates were used for in vitro enzyme activity assay and intrinsic tryptophan fluorescence experiments; this sample size was chosen as is typical in the literature for such experiments.
Data exclusions	No data were excluded from analyses.
Replication	All experiments could be replicated successfully.
Randomization	Protein samples and E. coli cultures were randomly assigned to control or experimental groups.
Blinding	This is not relevant to the study as the identify of the experimenters could not influence experimental results.

Reporting for specific materials, systems and methods

We require information from authors about some types of materials, experimental systems and methods used in many studies. Here, indicate whether each material, system or method listed is relevant to your study. If you are not sure if a list item applies to your research, read the appropriate section before selecting a response.

Materials & experimental systems

n/a	Involved in the study
☐	☒ Antibodies
☒	☐ Eukaryotic cell lines
☒	☐ Palaeontology and archaeology
☒	☐ Animals and other organisms
☒	☐ Clinical data
☒	☐ Dual use research of concern

Methods

n/a	Involved in the study
☒	☐ ChIP-seq
☒	☐ Flow cytometry
☒	☐ MRI-based neuroimaging

Antibodies

Antibodies used	Anti-FLAG M2 antibody (1:5000 dilution; Sigma-Aldrich, product number F3165-.2MG), secondary polyclonal Goat Anti-Mouse IgG H&L (HRP) antibody (1:5000 dilution; Abcam, Massachusetts, product number ab6789.
Validation	According to the Sigma-Aldrich product specification sheet (https://www.sigmaaldrich.com/specification-sheets/120/274/F3165-BULK.pdf) and the data sheet (https://www.sigmaaldrich.com/deepweb/assets/sigmaaldrich/product/documents/274/912/f3165dat-ms.pdf) for the monoclonal anti-flag M2 antibody produced in mouse, this antibody can detect 2 ng of FLAG-BP fusion protein by dot blot using chemiluminescent detection (sensitivity) and can detect a single band of protein on a western blot from an E. coli crude lysate (specificity).<br>The secondary goat anti-mouse IgG (HRP) was used according to AbCam's product data sheet (https://www.abcam.com/products/secondary-antibodies/goat-mouse-igg-hl-hrp-ab6789.html).
